# Determination of the native features of the exoglucanase Cel48S from *Clostridium thermocellum*

**DOI:** 10.1186/s13068-017-1009-4

**Published:** 2018-01-13

**Authors:** Ya-Jun Liu, Shiyue Liu, Sheng Dong, Renmin Li, Yingang Feng, Qiu Cui

**Affiliations:** 1 Shandong Provincial Key Laboratory of Energy Genetics, CAS Key Laboratory of Biofuels, Qingdao Engineering Laboratory of Single Cell Oil, Qingdao Institute of Bioenergy and Bioprocess Technology, Chinese Academy of Sciences, Qingdao, 266101 People’s Republic of China; 2 University of Chinese Academy of Sciences, Chinese Academy of Sciences, Beijing, 100049 People’s Republic of China

**Keywords:** Activity, Cellulosome, Crystalline cellulose, Exocellulase, Lignocellulose, Substrate specificity

## Abstract

**Background:**

*Clostridium thermocellum* is considered one of the most efficient natural cellulose degraders because of its cellulosomal system. As the major exoglucanase of cellulosome in *C. thermocellum*, Cel48S plays key roles and influences the activity and features of cellulosome to a great extent. Thus, it is of great importance to reveal the enzymatic features of Cel48S. However, Cel48S has not been well performed due to difficulties in purifying either recombinant or native Cel48S proteins.

**Results:**

We observed that the soluble fraction of the catalytic domain of Cel48S (Cel48S_CD) obtained by heterologous expression in *Escherichia coli* and denaturation-refolding treatment contained a large portion of incorrectly folded proteins with low activity. Using a previously developed seamless genome-editing system for *C. thermocellum*, we achieved direct purification of Cel48S_CD from the culture supernatant of *C. thermocellum* DSM1313 by inserting a sequence encoding 12 successive histidine residues and a TAA stop codon immediately behind the GH domain of Cel48S. Based on the fully active protein, biochemical and structural analyses were performed to reveal its innate characteristics. The native Cel48S_CD showed high activity of 117.61 ± 2.98 U/mg and apparent substrate preference for crystalline cellulose under the assay conditions. The crystal structure of the native GH48 protein revealed substrate-coupled changes in the residue conformation, indicating induced-fit effects between Cel48S_CD and substrates. Mass spectrum and crystal structural analyses suggested no significant posttranslational modification in the native Cel48S_CD protein.

**Conclusion:**

Our results confirmed that the high activity and substrate specificity of Cel48S_CD from *C. thermocellum* were consistent with its importance in the cellulosome. The structure of the native Cel48S_CD protein revealed evidence of conformational changes during substrate binding. In addition, our study provided a reliable method for in situ purification of cellulosomal and other secretive proteins from *C. thermocellum*.

**Electronic supplementary material:**

The online version of this article (10.1186/s13068-017-1009-4) contains supplementary material, which is available to authorized users.

## Background

Cellulosome is an extracellular multiprotein complex produced by cellulolytic bacteria. This macromolecular machine comprises numerous cellulases, nonenzymatic scaffoldins, and other dockerin-containing auxiliary proteins [1]. By organizing and concerting different functional components, the cellulosome makes full use of synergistic effects and shows apparent advantages in lignocellulose hydrolysis [2–4], especially toward crystalline cellulose compared with fungal cellulases [5]. Family 48 glucosyl hydrolases (GH48s) are exoglucanases that attack cellulose chains from the reducing ends to progressively release cellobiose molecule, thereby playing crucial roles in the hydrolysis of crystalline cellulose [6–9]. *Clostridium thermocellum* is known as the first-described cellulosome-producing and cellulolytic bacterium [10] and has potential use in lignocellulose bioconversion via consolidated bioprocessing [5, 11, 12]. *C. thermocellum* produces only one cellulosomal GH48 exoglucanase, termed Cel48S (also known as S_s_ or S8) [8, 13, 14]. Olson et al. investigated the contribution of Cel48S to the cellulolytic activity of *C. thermocellum* by gene deletion, and suggested that the lack of Cel48S could significantly reduce the hydrolysis rate of crystalline cellulose, thereby demonstrating the importance of the Cel48S enzyme in the cellulosome system [8, 15].

The Cel48S enzyme was first defined as an exoglucanase based on enzymatic analysis of the S8-tr component of the cellulosome [14]. Subsequently, the role of Cel48S as an exoglucanase was further confirmed by biochemical and structural studies based on recombinant proteins those were heterologously expressed in *E. coli* [16–20]. However, a relatively low activity or a substrate preference for amorphous cellulose instead of crystalline cellulose has been reported for this exoglucanase [16–18, 21, 22], which did not agree with its essential enzymatic role in cellulose degradation. Recombinant Cel48S or its catalytic domain (rCel48S_CD) proteins are primarily produced in the form of insoluble inclusion body. Denaturation and dialysis processes may lead to incorrect refolding of the protein and improper enzymatic activity. Thus, although the importance of Cel48S in cellulose degradation has been widely accepted, its properties have not been extensively analyzed. Verification is required to clarify its structure and enzymatic features. In addition, difficulties in expressing and purifying recombinant Cel48S protein have hindered further studies on the genetic modifications and site-directed mutations of Cel48S for higher activity or reduced feedback inhibition, i.e., features which have been proposed based on published crystal structures [19, 23, 24].

The Cel48S enzyme is the most abundant component in the extracellular cellulosome of *C. thermocellum* [25, 26] and provides the potential for purifying native Cel48S directly from the cellulosomal or extracellular proteins. However, the native Cel48S protein tightly binds to the scaffoldin protein CipA via strong interactions between the type I dockerin domain of Cel48S and type I cohesin domain of CipA [27]. This high affinity greatly prevents the liberation of Cel48S and other functional components to ensure the stability of the quaternary structure of the cellulosomal complex [28]. Morag et al. reported that the S8-tr component could be isolated from the cellulosomal complex by protease K digestion [14]. Purified S8-tr was slightly smaller than the S8 component in the cellulosome, indicating that S8-tr may be a truncated Cel48S protein without the type I dockerin module [14]. Thus, purification of the catalytic domain of Cel48S can be achieved by releasing the protein from the type I dockerin–cohesin interaction. By using a previously developed seamless genome editing system, a stop codon could be inserted in front of the dockerin module in the chromosome to extracellularly release the catalytic domain of Cel48S. In addition, affinity tags could be fused to the target protein to simplify the purification process and improve purification quality.

## Methods

### Bacterial strains and cultivation

Bacterial strains used in this study are listed in Table 1. *Escherichia coli* strains were cultivated aerobically at 37 °C in Luria–Bertani (LB) liquid medium with shaking at 200 rpm or on solid LB plate with 1.5% agar. *C. thermocellum* strains were grown anaerobically at 55 °C in modified GS-2 [29] or MJ medium [30] with 5 g/L cellobiose or Avicel (PH-101, Sigma) as the carbon source unless otherwise stated. 30 μg/mL chloramphenicol, 50 μg/mL kanamycin, and 3.3 μg/mL thiamphenicol (Tm) were supplemented to the medium when necessary. 10 μg/mL 5-fluoro-2-deoxyuradine (FUDR) or 500 μg/mL 5-fluoroorotic acid (FOA) dissolved in dimethyl sulfoxide were added for screening.

**Table 1:** Bacterial strains and plasmids used in this study

Strains/plasmids	Relevant characteristic	Sources
Strains		
*E. coli*		
DH5α	*f80dlacZΔM15, Δ(lacZYA*-*argF)U169, deoR, recA1, endA1, hsdR17(rk* − *, mk* +*), phoA, supE44, l* − *, thi*-*1, gyrA96, relA1*	Transgen
BL21(DE3)	*ompT gal dcm lon hsdSB(rB* − *mB* −*) l (DE3 [lacI lacUV5*-*T7 gene 1 ind1 sam7 nin5])*	Transgen
BL21(DE3)::pET28aNS-rCel48S_CD	Derived from BL21(DE3), carrying plasmid pET28aNS-rCel48S_CD to express the recombinant Cel48S_CD (rCel48S_CD)	This study
*C. thermocellum*		
ATCC27405	Wild type stain	ATCC
DSM1313	Wild type stain	DSMZ
*ΔpyrF*	Derived from DSM1313, with deleted *pyrF* gene	[32]
*ΔpyrF*::Cel48S_CD-His_12_	Derived from *ΔpyrF*, expressing the native Cel48S_CD bearing a His_12_ tag (ctCel48S_CD -His_12_)	This study
Plasmids		
pET28aNS	Expression vector with N-terminal hexahistidine affinity tag, with modified multiple cloning sites	[31]
pET28aNS-rCel48S_CD	pET28aNS derivative for expression of Cel48S_CD from *C. thermocellum* ATCC27405	This study
pHK-HR-*Ca*BglA	Markerless genome editing plasmid for DSM 1313	[32]
pHK-HR-His_12_TAA	pHK-HR-*Ca*BglA derivative for markerless insertion of His_12_TAA in the chromosome of DSM 1313	This study

### Construction of plasmids

Plasmid pET28NS-rCel48S_CD was constructed by cloning the catalytic domain of Cel48S (PDB 1L1Y) into pET28NS [31] using NheI and SpeI restriction sites (Table 1). Primers rCel48S-F/R were used for gene amplification with the genome DNA of *C. thermocellum* ATCC27405 as the templates (Additional file 1). To construct the plasmid pHK-HR-His_12_TAA for markerless insertion of 12-histidine-encoding sequence (His_12_) as well as a stop codon TAA (His_12_TAA) into the chromosome of *C. thermocellum* DSM1313, the double-strand His_12_TAA sequence was created by annealing reverse complementary primers H_12_TAA-1/2 at room temperature to replace the *Ca*BglA sequence of pHK-HR-*Ca*BglA by EagI and MluI digestion (Table 1, Additional file 1).

### Expression and purification of recombinant and native Cel48S_CD proteins

Plasmid pET28NS-rCel48S_CD was transformed to *E.coli* BL21(DE3). The transformant was cultured in LB medium at 37 °C for 3 h when the optical density at 600 nm reached 0.8–1.0. The synthesis of recombinant Cel48S_CD (rCel48S_CD) proteins in BL21(DE3)::pET28NS-rCel48S_CD was initiated by the addition of 1 mM IPTG. Cultivation was continued for another 16 h at 16 °C. Cells were harvested by centrifugation at 10,000 g and disrupted by sonication in an ice-water bath. The rCel48S_CD protein was primarily expressed as insoluble inclusion bodies as previously reported [16, 17]. The inclusion bodies were dissolved in 50 mM Tris–HCl buffer (pH 8.0) containing 30 mM imidazole, 500 mM NaCl, and 8 M urea—and the supernatant was used for protein purification after centrifugation at 20,000*g* for 20 min at 4 °C and subsequent microfiltration (0.22-μm filter). Ni^2+^-affinity chromatography was used for the first-step purification because the hexahistidine cascade of pET28NS was fused to the *celS* gene at its 5′ end during plasmid construction. After purification, the rCel48S_CD protein was dialyzed against 10 mM Tris–HCl buffer (pH 8.0) containing 100 mM NaCl for 4–9 h at 25 °C with two to three buffer changes. A final purification step of rCel48S_CD was carried out by gel filtration using a Superdex 200 column (GE Healthcare).

Native Cel48S_CD (ctCel48S_CD) proteins were directly purified from the culture broth of *C. thermocellum* DSM 1313. Cells were first cultivated in GS-2 medium with 5 g/L Avicel as the carbon source for 40 h until the late exponential phase. Then, the supernatants were harvested by centrifugation at 20,000*g* for 20 min at 4 °C and subsequent microfiltration (0.22-μm filter). Supernatant extracellular proteins were further used for ctCel48S_CD purification using a Ni^2+^ Sepharose HP column (GE healthcare) and, subsequently, a Superdex 200 column (GE Healthcare). All purified Cel48S_CD proteins were conserved at − 80 °C for further analysis.

### Seamless genome editing of *C. thermocellum*

Seamless insertion of His_12_TAA sequence in the chromosome of *C. thermocellum* DSM1313 was performed through two rounds of homologous recombination, as previously described [32]. In brief, after the transformation of plasmid pHK-HR-His_12_TAA, the transformants were selected on Tm, and inoculated into uracil auxotrophic MJ medium in the presence of FUDR to promote the first round of homologous chromosomal integration and the elimination of transformed plasmid. Then, positive colonies verified by PCR (~ 4.4 kb) were plated on FOA-added GS-2 medium to remove the *pyrF* cassette through the second round of recombination. Colony PCR and sequencing were subsequently performed to identify the insertion of His_12_TAA (Fig. 2b).

### SDS-PAGE and protein analyses

Sodium dodecyl sulfate polyacrylamide gel electrophoresis (SDS-PAGE) was performed to check protein purity and composition as previously described [32] using QuickRun buffer (MDbio) and protein standards ranging from 10 to 245 kDa or from 14 to 116 kDa (New England BioLabs). Protein quantification was performed using the Bradford method before further analyses [33]. Protein molecular weight was determined using a high-performance liquid chromatography (HPLC) system (Agilent 1290) coupled to a Q-TOF–MS (Agilent 6530). The HPLC was equipped with a Zorbax 300SB-C8 column (4.6 × 250 mm, Agilent). Samples were run at a flow rate of 1 mL/min with 6-μL injection volume. The mobile phases contained 0.1% (vol/vol) formic acid in water (A) or in acetonitrile (B). A four-step linear gradient of 5% B from 0 to 5 min, 5–50% B from 5 to 10 min, 50–90% B from 10 to 12 min, and 90% B from 12 to 15 min was used. The scan range was *m*/*z* 800–1800. The deconvolution analysis was displayed by the software Deconvolute (MS):protein (Agilent).

### Circular dichroism (CD) analyses

CD spectra were acquired using a J-715 spectrapolarimeter (JASCO). Protein samples were dialyzed against PBS buffer (pH 7.4) and diluted to 0.1 mg/mL prior to measurements. Wavelength spectra were recorded at 25 °C with a path length of 0.5 cm. Each scan was obtained by recording every 0.5 nm between the wavelength ranges of 200–250 nm. The mean residue ellipticity units (MRE, deg cm^2^ dmol^−1^ per residue) were calculated for analysis.

### Tryptophan intrinsic fluorescence (TIF) analyses

TIF analyses were performed for the same protein samples using a fluorescence spectrophotometer F-4600 (Hitachi) at 25 °C, as those used for the CD analysis. The path length was 0.1 cm. Excitation wavelength was 295 nm, and the emission spectra were recorded from 310 to 490 nm (1-nm bandwidth) as the fluorescence intensity [34].

### Cellulase assay

The Cel48S activity was determined by analyzing reducing sugars released from Avicel (PH-101, Fluka), phosphoric acid-swollen cellulose (PASC, prepared from Avicel as described previously) [35], carboxymethyl cellulose-Na (CMC, Sigma), xylan (Mucklin), pectin (aladdin), cellobiose, cellotriose, cellotetrose, or cellopentose (Megazyme) by HPLC as previously described [36]. The enzyme activities were measured after incubation at 55 °C in 50 mM succinate buffer (pH 5.7) containing 10 mM CaCl_2_ and 1% (wt/vol) substrate mentioned above for 5 h, unless otherwise stated. 10 μg purified enzyme was added to the system to initiate the reaction. One unit of enzyme activity is defined as the amount of enzyme that releases 1 nmol reducing sugar (glucose equivalent) per min.

### Kinetic characterization

The reaction time with initial rate of Cel48S_CD was investigated in prior to kinetic characterization by measuring enzyme activity after reacting for 0.5–24 h. Nonlinear kinetic analyses were performed at 55 °C based on initial rates determined from 1 to 10 mg/mL of Avicel and analyzed by the Michaelis–Menten equation using the OriginPro 8.5.1 SR2 software. Avicel was also used to determine the optimal conditions of the Cel48S_CD proteins.

### Crystallization, data collection, structural determination, and refinement

The ctCel48S_CD proteins (~ 12 mg/mL) in 10 mM Tris–HCl buffer including 100 mM NaCl were crystallized by using commercial high throughput screening kits (Hampton Research) and the sitting drop vapor diffusion method. The crystallization conditions able to generate crystals were further optimized in 24-well crystallization plates. The crystals used for X-ray data collection were obtained in 200 mM sodium formate with 20% PEG3350. All crystals were cryoprotected by soaking in well solution supplemented with 30% (v/v) glycerol for 10 s and then flash cooled to 100 K in liquid nitrogen. The data were collected at the Shanghai Synchrotron Radiation Facility, Beamline BL17U, in a 100 K nitrogen stream [37]. Data indexing, integration, and scaling were conducted using MOSFLM [38]. The ctCel48S_CD structure was determined by molecular replacement using CCP4 program suit and the PDB file 1L2A as a search model [19]. Refinement of the structure was performed using the programs COOT and PHENIX [39, 40]. The final model was evaluated using PROCHECK. All molecular graphics were created using PyMOL v1.8 (http://www.pymol.org).

## Results

### Purification and enzymatic analysis of recombinant Cel48S_CD

**Fig. 1:**
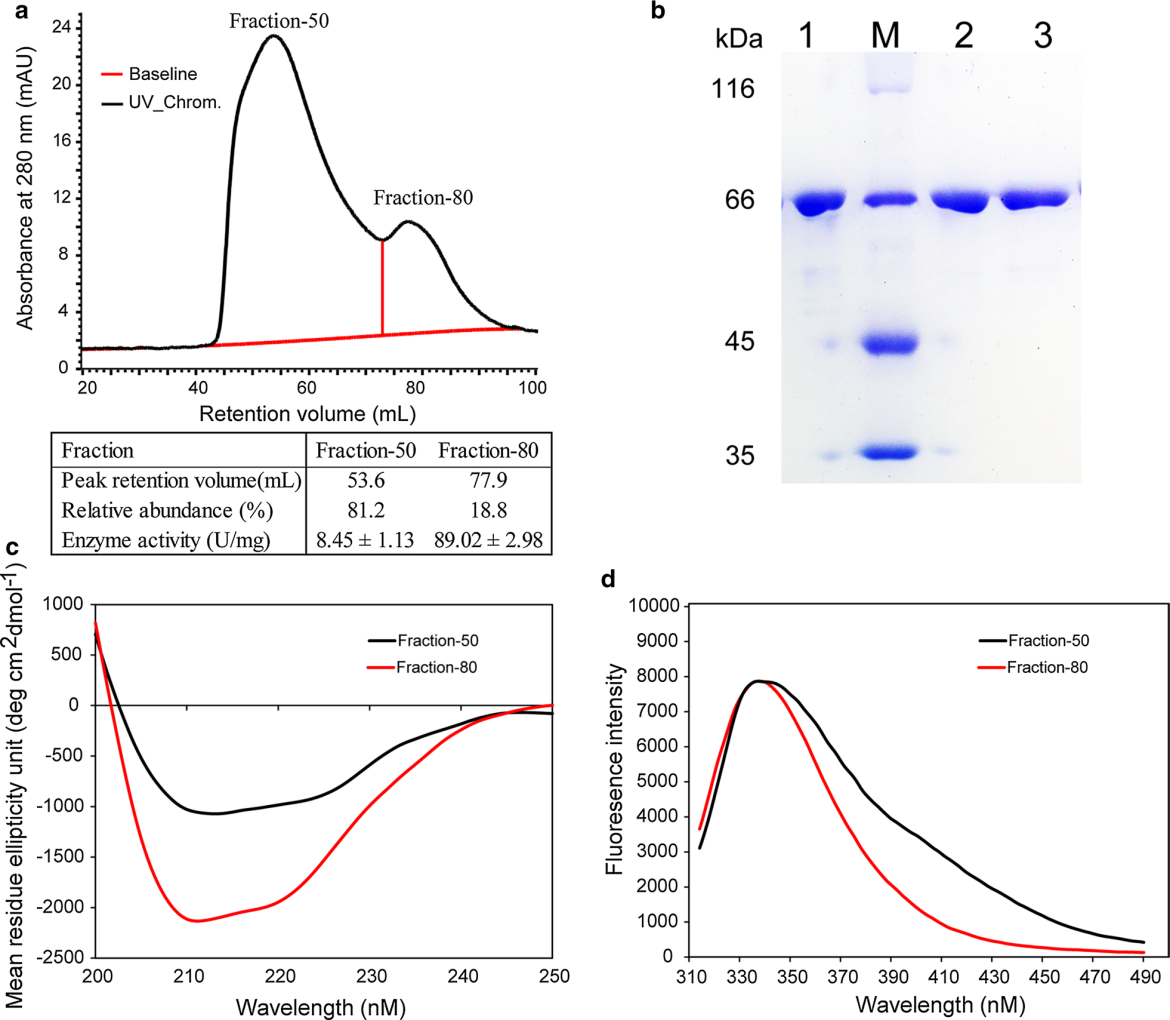
Purification and analyses of rCel48S_CD proteins. **a** Purification of the rCel48S_CD protein by gel filtration. After Ni^2+^-affinity purification, denatured rCel48S_CD proteins were dialyzed against 10 mM Tris–HCl buffer (pH 8.0) containing 100 mM NaCl for 4 h at 25 °C with two buffer changes, and were further purified using a Superdex 200 column (GE healthcare). Two peaks were detected with retention volumes of approximately 50 and 80 mL (fraction-50 and fraction-80, respectively). The relative abundances of fraction-50 or fraction-80 were determined by calculating the proportion of the areas of corresponding peaks in the total area, i.e., the relative abundance of fraction-50 is Area_fraction-50_/(Area_fraction-50_ + Area_fraction-80_). The activities of each fraction are shown below the chromatogram. **b** SDS-PAGE analysis. Lane 1, rCel48S_CD protein after Ni^2+^-affinity purification; lane 2, fraction-50; lane 3, fraction-80. M, protein standards. **c** CD analyses. CD spectra are given as the standard mean residue ellipticity units (MREs). **d** TIF analyses. The emission spectra from 314 to 490 nm were detected after excitation at 295 nm. The absorption spectrum of fraction-50 is red-shifted slightly and has reduced slope with that of fraction-80, indicating difference in the secondary structures [55]

Heterologous expression of rCel48S_CD in *E. coli* BL21(DE3) was performed. The obtained rCel48S_CD was mainly in the form of inclusion bodies, which was consistent with published reports [16]. The denaturation step was carried out as previously described. Instead of ion-exchange chromatography [17], we used Ni^2+^-affinity chromatography for protein purification because the expressed protein had an N-terminal His_6_-tag. The eluted protein was dialyzed against 10 mM Tris–HCl buffer (pH 8.0) containing 100 mM NaCl for 4 h at 25 °C with two buffer changes and was used to analyze the cellulase activity. The result indicated that the obtained rCel48S_CD had an activity of 17.54 U/mg using Avicel as the substrate, which was similar to or higher than that reported in the literature [16, 22]. The protein was further purified by gel filtration. Two fractions were detected with retention volumes of approximately 50 and 80 mL (fraction-50 and fraction-80, respectively) (Fig. 1a). Sodium dodecyl sulfate polyacrylamide gel electrophoresis (SDS-PAGE) analysis indicated that both fractions had the expected size of rCel48S_CD (72.8 KDa) (Fig. 1b). However, the cellulase activity of the fraction-50 was only 8.45 ± 1.13 U/mg, which was 9.5% of that of the fraction-80 (89.02 ± 2.98 U/mg). Both circular dichroism (CD) spectra and tryptophan intrinsic fluorescence (TIF) analyses showed that fraction-50 and fraction-80 presented differences in their secondary structures (Fig. 1c, d), indicating that fraction-50 may be an incorrectly folded oligomer of rCel48S_CD. The relative abundance of the two fractions in the eluted protein was estimated by calculating the proportion of the areas of the corresponding peaks. This result suggested that, after Ni^2+^-affinity purification, 81.2% of the proteins were in fraction-50 with low activity (Fig. 1a).

To promote the refolding of rCel48S_CD, three buffer changes (2 h each) were performed during dialysis to remove as much urea as possible. Gel filtration was performed to detect the changes in fraction-50 and fraction-80 (Additional file 2). The result indicated that the proportion of fraction-50 decreased to 35.8%. The activities of fraction-50 and fraction-80 increased to 10.40 and 95.49 U/mg, respectively (Additional file 2). The dialysis duration was further extended to 3 h per dialysis, resulting in a further reduction of fraction-50 (27.6%). The activity of fraction-80 was also enhanced to 105.91 U/mg with this treatment (Additional file 2). Although a sixfold higher activity of rCel48S_CD was obtained after the optimization of the purification process, the fraction-50 peak was yet to be detected, and the recombinant protein’s quality and activity were easily influenced by the purification process. In addition, fraction-80 may have still contained proteins that belonged to fraction-50. This result demonstrated that a complicated purification process of multiple steps was required for purification of the recombinant protein. Thus, characterization of the CelS protein should not rely on the heterologously expressed recombinant protein purified using the current procedure.

### Construction of a *C. thermocellum* recombinant strain producing the catalytic domain of ctCel48S_CD with histidine-tag

**Fig. 2:**
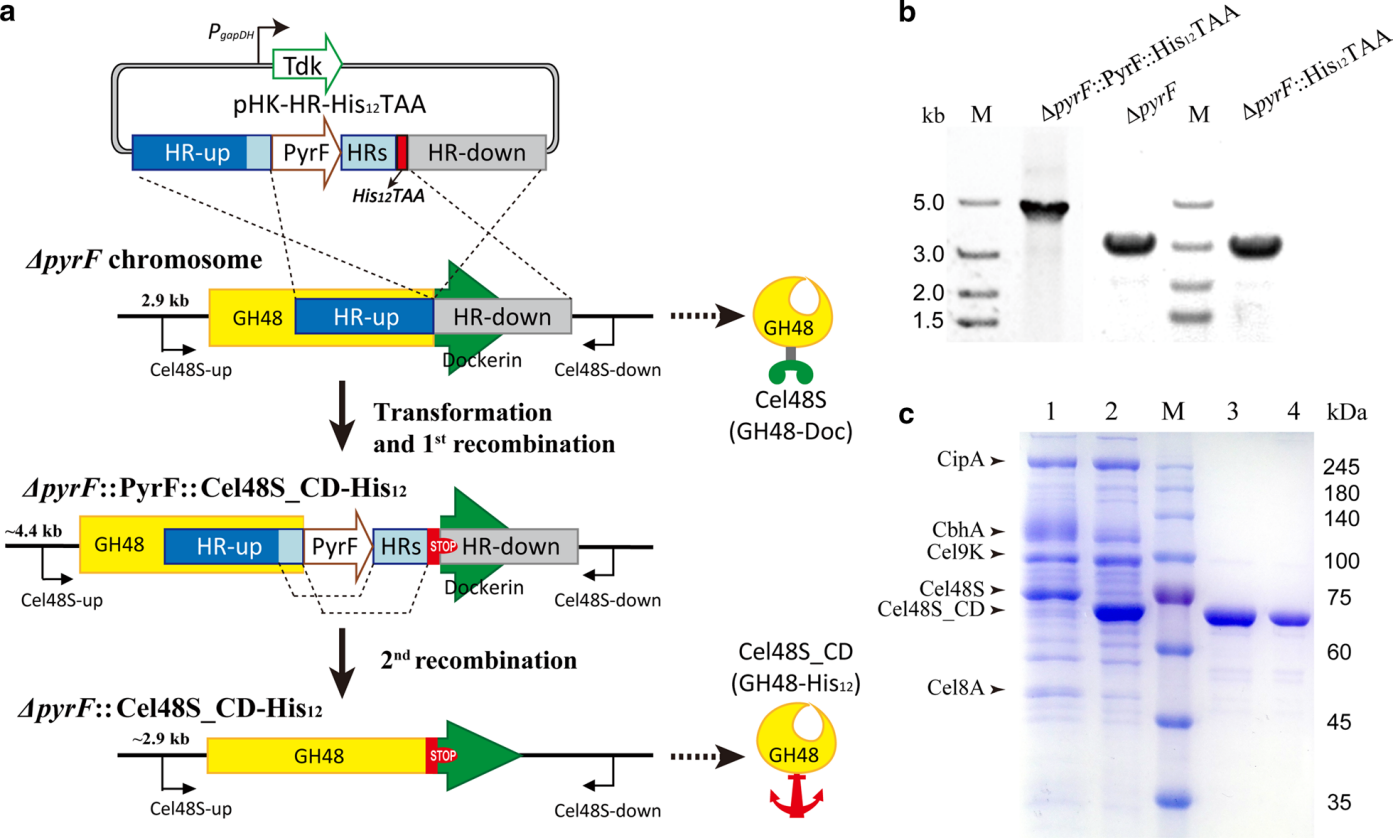
Knock–in of a sequence encoding 12 successive histidine residues (His_12_) and a TAA stop codon in the chromosome of *C. thermocellum* ∆*pyrF*. **a** Schematic representation of the workflow of the seamless genome-editing system using the plasmid pHK–HR–His_12_TAA. The first round of recombination used the combined selection of FUDR toward Tdk and uracil auxotrophic medium toward PyrF after transformation of the plasmid into ∆*pyrF* strain. The second round of recombination occurred in the presence of the FOA selection stress. The *pyrF* cassette was removed. Compared with the parent strain *∆pyrF*, the target mutant *∆pyrF*::Cel48S_CD-His_12_ produced the Cel48S_CD protein containing a His_12_-tag (GH48-His_12_), instead of the type I dockerin (GH48-Doc). **b** Diagnostic PCR investigation of the mutant strain after each recombination step. M, DNA marker. **c** SDS-PAGE analysis of the extracellular proteins of *∆pyrF* (Lane 1) and ∆*pyrF*::Cel48S_CD-His_12_ (Lane 2) strains. The supernatants were tenfold condensed (10–1 mL) by ultrafiltration (10.0 kDa cutoff) (Merck Millipore, Billerica), and approximately 5 μg of the protein samples was loaded in each lane. The bands corresponding to known cellulosomal proteins are identified to the left of the Coomassie blue-stained gel. The extracellular proteins of ∆*pyrF*::Cel48S_CD-His_12_ was purified by Ni^2+^ affinity chromatography and subsequently, by gel filtration. Lanes 3 and 4 show the SDS-PAGE analysis of the eluted fractions, respectively. M, protein standards

The type I cohesin–dockerin interaction has a dissociation constant of over 10^9^ M in the presence of calcium. Because of the strong interactions, the cellulosomal system maintains stable structures and resists environmental interference [2]. Thus, isolation and purification process of individual components with activity from the cellulosomal complex is not easy to achieve [41, 42]. To free the catalytic domain from the type I interaction, we inserted a sequence encoding 12 successive histidine residues (His_12_) and a TAA stop codon into the genome located between the catalyzing module- and dockerin module-encoding regions of Cel48S by means of a previously developed seamless genome-editing system [32]. The inserted TAA stop codon terminates protein translation ahead of the dockerin-encoding sequence. The expressed Cel48S catalyzing domain contains a His_12_-tag at its C-terminal for affinity purification (Fig. 2a). The plasmid pHK-HR-His_12_TAA was constructed by replacing the CaBglA sequence of pHK-HR-*Ca*BglA [32] with a (CAT)_12_TAA sequence and was subsequently transformed into the PyrF-deleted *C. thermocellum* DSM1313 strain *∆pyrF* [32]. The target mutant *∆pyrF*:: Cel48S_CD-His_12_ was obtained after two rounds of recombination as previously described [32] and was verified by PCR and sequencing (Fig. 2a, b).

### Expression and purification of the native ctCel48S_CD

**Fig. 3:**
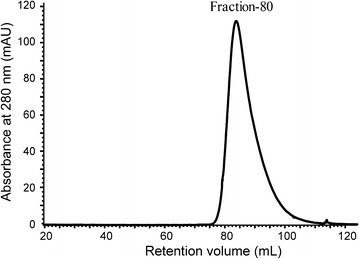
Gel filtration chromatogram of ctCel48S_CD protein. The soluble ctCel48S_CD protein was purified using Ni^2+^-affinity and subsequently, by gel filtration chromatography. No fraction-50 was detected in the gel filtration chromatogram

Both *∆pyrF* and *∆pyrF*::Cel48S_CD-His_12_ were cultivated in GS-2 medium with 5 g/L Avicel as the carbon source for 40 h. Culture supernatants were harvested as extracellular proteins and were tenfold condensed by ultrafiltration for SDS-PAGE analysis (Fig. 2c). Compared with the parent strain, the mutant strain *∆pyrF*::Cel48S_CD-His_12_ produced a truncated, smaller sized Cel48S protein, indicating the exclusion of the dockerin module, as expected. The ctCel48S_CD protein produced by *∆pyrF*::Cel48S_CD-His_12_ contained a C-terminal His_12_-tag (Fig. 2a, Additional file 3), thereby was directly purified from the extracellular proteins using Ni^2+^-affinity chromatography. Gel filtration chromatography was subsequently performed for further purification using a Superdex 200 column. In contrast to rCel48S_CD (Fig. 1a), the gel filtration chromatogram of ctCel48S_CD showed only one peak with a retention volume of 83 mL, which indicated that the ctCel48S_CD protein was only in a monomeric form (Fig. 3). In addition, the SDS-PAGE result suggested that the ctCel48S_CD protein already had high purity after the one-step affinity purification; gel filtration was not necessary (Fig. 2c).

The molecular weight (MW) of the purified ctCel48S_CD protein was analyzed by HPLC-Q-TOF–MS (Additional file 3). The results showed that the MW of ctCel48S_CD was 74,271.62 Da, which is similar to its theoretical value 74267.25 Da which was calculated based on the sequence of GH domain of Cel48S and a His_12_-tag, indicating no posttranslational modifications.

### Enzymatic characterization of ctCel48S_CD

**Fig. 4:**
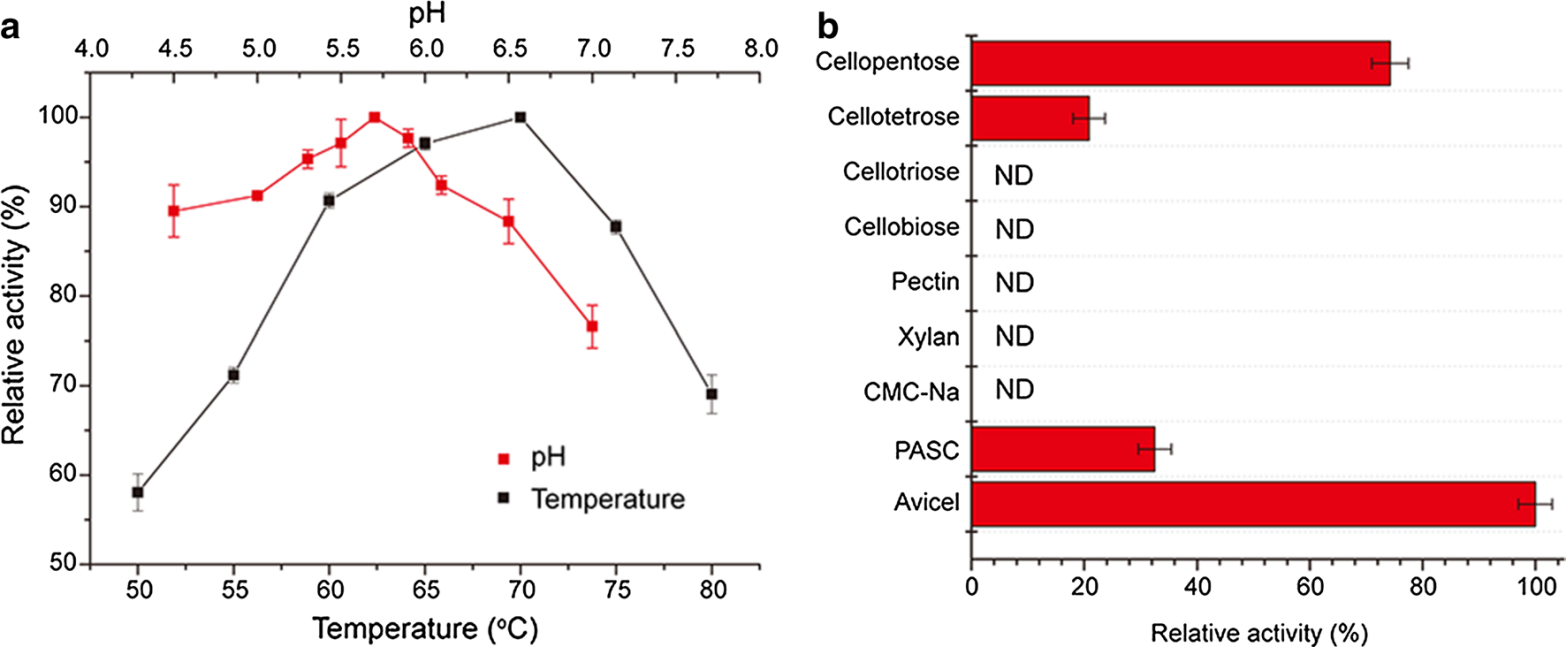
Enzymatic characterization of the ctCel48S_CD proteins. **a** Effects of temperature and pH on ctCel48S_CD activity. The activities determined at 70 °C or pH 5.7 were considered as 100% for calculation. **b** The substrate specificity of ctCel48S_CD. The activity of ctCel48S_CD was determined using different substrates, and the activity toward Avicel was considered as 100% for calculation. ND, not detected

Using Avicel as a substrate, the specific activity of ctCel48S_CD was determined to be 117.61 ± 2.98 U/mg, which was 1.11-fold greater than the highest activity detected for rCel48S_CD fraction-80 in this study. The optimal conditions were analyzed under the assay conditions, and ctCel48S_CD showed the highest activity at 70 °C and pH 5.7 (Fig. 4a), which is consistent with rCel48S_CD [17]. To mimic the optimal conditions of the host *C. thermocellum* and the cellulosome [43], the substrate specificity of ctCel48S_CD was determined by measuring the activity at 55 °C and pH 5.7 for various substrates, including Avicel, CMC, PASC, cellotetrose, cellotriose, cellopentose, cellobiose, pectin, and xylan (Fig. 4b). The ctCel48S_CD protein showed the highest activity when using Avicel as the substrate, and the activity on PASC or CMC-Na was relatively low or not detected. ctCel48S_CD could also hydrolyze cellopentose and cellotetrose, but no activity was detected for cellobiose or cellotriose. In addition, ctCel48S_CD showed no activity with pectin or xylan serving as the substrate, which is consistent with previous data [17].

The reaction period initial rate was determined prior to the kinetic analysis because a relatively long reaction time (5 h) was used in a previous study [17]. The results showed a linear relationship between the reaction time and glucose concentration within 6 h, indicating that the catalyzing reaction maintained the initial rate over the same period (Additional file 4). Hence, the reaction time was determined to be 5 h in further kinetic analysis. For an Avicel concentration range of 1–10 mg/mL, the *Km* value of ctCel48S_CD was determined to be 6.84 mM using nonlinear analysis according to the Michaelis–Menten equation (Additional file 4).

### Crystallization and structural analysis of ctCel48S_CD

The native ctCel48S_CD protein and the previously reported recombinant protein rCel48S_CD showed different substrate preferences, i.e., ctCel48S_CD showed higher activity toward crystalline cellulose (Fig. 4b), whereas rCel48S_CD had higher activity toward amorphous cellulose [17]. Because the crystal structure of rCel48S_CD has been reported [19], crystallization and structural analysis were also performed for ctCel48S_CD to investigate potential differences.

**Fig. 5:**
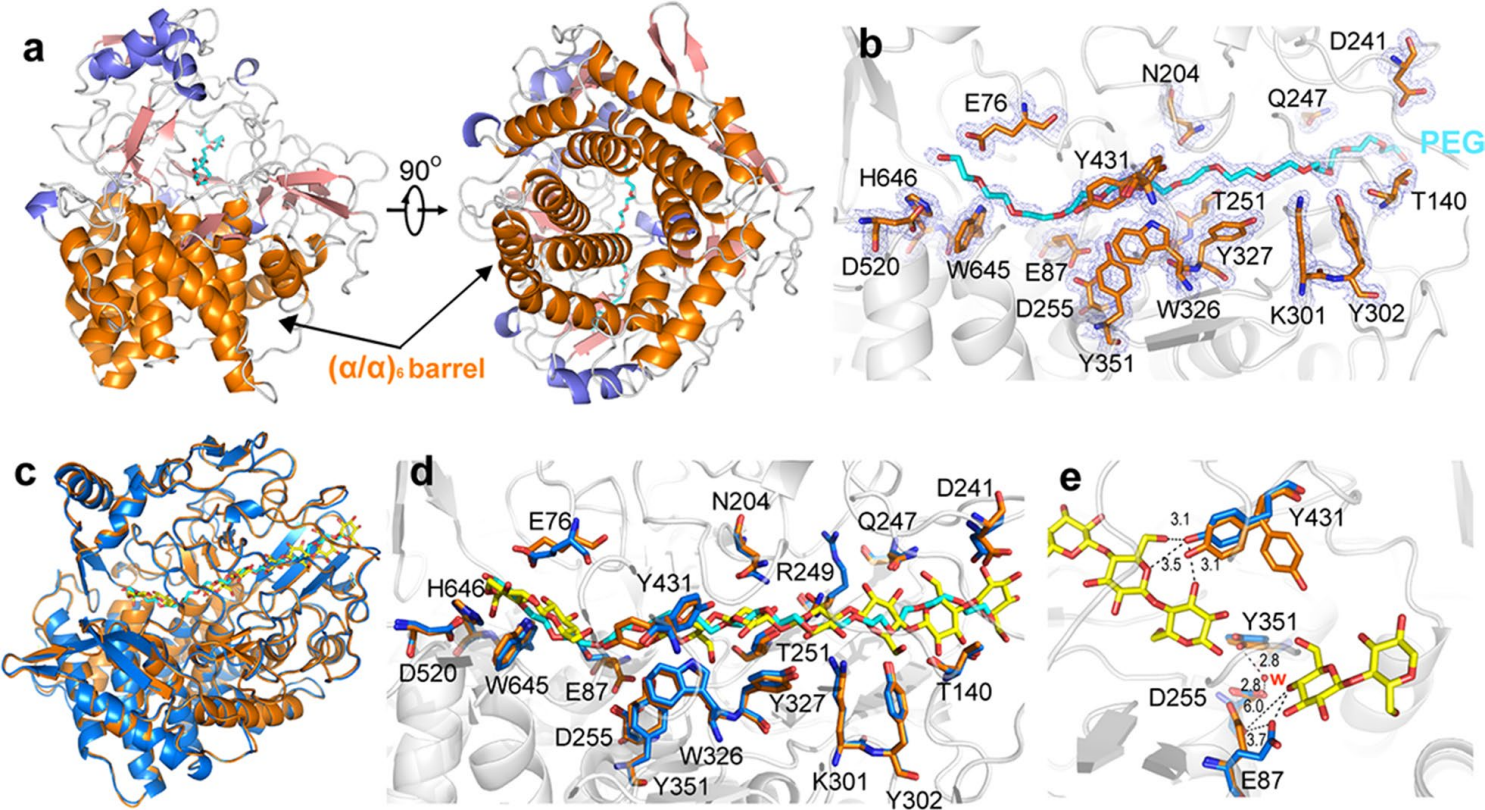
Structural analysis of ctCel48S_CD from *C. thermocellum* DSM1313. **a** The overall structure of ctCel48S_CD in complexation with PEG. The core (α/α)_6_ barrels are colored in orange, the additional α-helixes in slate, and β-sheets in pink. **b** The substrate-binding tunnel of ctCel48S_CD with a bound PEG. PEG is in cyan, and residues that distribute in the catalytic tunnel are shown in blue sticks. The 2*F*_*o*_*F*_*c*_ densities for PEG and residues are contoured in blue in 1.0σ. **c** Superimposed structures of ctCel48S_CD and rCel48S_CD. The rCel48S_CD structure (1L2A) is in blue, and the oligosaccharides are in yellow. The ctCel48S_CD is in orange, and the bound PEG is in cyan. **d** Superposition of the residues responsible for substrate binding and catalysis. Residues of rCel48S_CD and ctCel48S_CD are in blue and orange, respectively. **e** The analysis of the conformationals change of residues in the catalytic center. The oligosaccharides are in yellow. The rCel48S_CD and ctCel48S_CD structures are in blue and orange, respectively. The possible hydrogen bonds are represented by dash line, and the distances are shown in angstroms

The crystal structure of the purified ctCel48S_CD protein was determined at 1.43Å obtaining final *R*_work_ and *R*_free_ values of 0.1601 and 0.1930, respectively. Data collection and refinement statistics are provided in Table 2. The coordinates and structure factors were deposited in the Protein Data Bank (PDB) under the accession number 5YJ6. The crystal structure showed one molecule in an asymmetric unit. The ctCel48S_CD protein adopted a typical (α/α)_6_ consisting of an inner core of six mutually parallel α-helixes connected by long loops, additional helices, or sheets to the six peripheral α-helixes (Fig. 5a). Unlike the published rCel48S_CD structures that contain a cellohexaose or a cellobiose molecule in the substrate-binding tunnel (PDB accession number 1L2A or 1L1Y, respectively) [19], the crystal structure of ctCel48S_CD was not in complex with any substrates or products, and a PEG molecule was bound instead (Fig. 5b). Interestingly, the PEG molecule in the ctCel48S_CD structure had a similar arrangement with the cello-oligosaccharide molecule in the rCel48S_CD structure, but with completely different shapes (Fig. 5c, d). However, the electron density of PEG at the open cleft was not quite clear, indicating a weak interaction between the enzyme and PEG in this region.

**Table 2:** Data collection and refinement statistics

Parameter	
Data collection	
Space group	P 21 21 2
*a,b,c* (Å) α,β,γ	96.00, 101.07, 58.98 90.00, 90.00, 90.00
Wavelength	0.979
Resolution (Å)	101.07–1.43 (1.47–1.43)^a^
Unique reflections	100,821 (5353)
Completeness (%)	94.9 (69.2)
Redundancy	7.3 (6.8)
*Mean I/sigma* (*I*)	16.0 (2.3)
R_merge_^b^	0.069 (0.786)
Refinement	
*R*_*work*_*/R*_*free*_ (%)	16.01/19.30
No. atoms	
Proteins	5182
Ligands	40
waters	834
B-factors	
Average B-factor	21.00
Proteins	19.00
Ligands	49.50
Solvent	32.30
r.m.s.d.	
Bond length (Å)	0.017
Bond angles	1.601
Ramachandran statistics	
Favored (%)	98
Outliers (%)	0

^a^Numbers in parentheses refer to data in the highest resolution shell

^b^R_merge_ = Σ_*hkl*_Σ_*i*_|*I*(*hkl*)_*i*_- < *I(hkl)* > |/Σ_hkl_Σ_i_ < *I(hkl)*_*i*_>

The ctCel48S_CD structure was well matched with the published rCel48S_CD structure, with a C^α^ RMSD of approximately 0.32 Å (Fig. 5c), especially for the residues responsible for substrate binding (i.e., T140, Q247, K301, Y302, W326, Y327, Y351, D520, W645, and H646). However, a few residues showed conformational differences, such as E87, N204, R242, and Y431 (Fig. 5d). The side chain of residue N204 in the ctCel48S_CD structure only showed a slight conformation difference from that of rCel48S_CD; residue Y431 shows two conformations [chi1 dihedral angle were in g + (63.5°) and g − (− 72.7°) conformations, respectively], wherein one was similar to that in rCel48S_CD (chi1 was − 56° in g- conformation for PDB 1L1Y) and the other was quite different. The side chain of residue R249 in the ctCel48S_CD structure showed apparent conflicts with the substrate based on the published rCel48S_CD-oligosaccharide complex structure. This suggested that a conformational change of R249 occurs upon substrate binding. The side chain of E87 deviated approximately 3.7 Å from that of the substrate-bound rCel48S_CD, which resulted in an increasing distance (from 2.8 Å to 6.0 Å) between the carboxylate oxygen of E87 and C4 hydroxyl of glucosyl residues at subsite + 1 (Fig. 5e). Y431 was predicative for substrate binding by forming hydrogen bond with hydroxyl or carboxyl groups of the substrate. R249 may be involved in substrate binding, and E87 is the catalytic acid residue that is responsible for glycosidic bond hydrolysis. In view of these functions, the conformations of Y431, R249, and E87 in the ctCel48S_CD structure seemed less appropriate than those in rCel48S_CD. Because the ctCel48S_CD structure was substrate- and product-free compared with the published rCel48S_CD structure, these results indicated that the residue conformation of the Cel48S protein may differ as the substrate-binding status changes, indicating that induced-fit effects may occur between the enzyme and substrates.

## Discussion

*C. thermocellum* is considered to be one of the most promising candidates for lignocellulose bioconversion, and has been applied as a whole-cell catalyst for cellulose saccharification [32, 44]. The high efficiency in lignocellulose deconstruction of *C. thermocellum* is primarily due to its unique cellulosome system. Cellulosome is a multi-protein complex comprising various enzymatic and nonenzymatic subunits. All of the subunits are assembled together via noncovalent and specific protein interactions between dockerin and cohesin domains [2, 4]. Protein–protein, protein–cell, and protein–substrate interactions in the cellulosomal system result in strong synergistic effects, which greatly enhance the cellulose hydrolytic activity of the cellulosome [22, 45–47]. Compared with fungal cellulases, the cellulosome shows apparent advantage in the degradation of crystalline cellulose [5] because it contains exocellulases that are capable of completely solubilizing crystalline forms of cellulose [27].

Morag et al. first isolated the cellulosomal S8-tr component and analyzed its enzymatic properties [14]. The S8-tr component mainly comprised the Cel48S_CD protein because they had similar molecular weights and properties. Thus, this was the first time that Cel48S was shown to be an exoglucanase [14]. Subsequently, Cel48S was defined as a family 48 cellulase because it contains a GH48-catalyzing domain with reducing end-acting cellobiohydrolase activity and a type I dockerin domain for assembly [19, 48]. The key role of Cel48S in the degradation of crystalline cellulose has been further verified by in vivo deletion in *C. thermocellum* and in vitro cellulosomal reconstitution or construction of designer cellulosomes [15, 22, 49].

Cel48S is the most abundant subunit in the cellulosome of *C. thermocellum*, and makes large contributions for determining the features of the whole cellulosomal system, especially in feedback inhibition and catalytic activity [32]. However, the enzymatic features of Cel48S have not been extensively analyzed. Most previous studies have primarily been performed based on the expression of recombinant Cel48S or its catalytic domain proteins and subsequent purification in *E. coli* as insoluble inclusion bodies. Relatively low activity has been reported, especially toward crystalline cellulose compared with amorphous cellulose [16–18, 21]. A recent study on the reconstitution of *C. thermocellum* cellulosome obtained the Cel48S protein using a cell-free protein synthesis and purification approach; even lower activity was detected [22]. Although a larger synergy effect between Cel48S and two other endoglucanases was observed for Avicel, the substrate specificity of Cel48S was considered for amorphous cellulose instead of Avicel [22]. The reported lower activity and preference for amorphous cellulose of Cel48S conflicted with its essential role in the hydrolysis of crystalline cellulose. Thus, the enzymatic features of Cel48S should be thoroughly characterized based on well-expressed and purified protein.

The difficulty of heterologous expression of soluble Cel48S protein has been verified in several studies. Thus, inclusion body proteins were used for biochemical and structural analyses after denaturation and dialysis [16–19]. However, the tedious and complicated treatment may cause protein degradation and incorrect or low-quality protein refolding. We expressed and purified the recombinant Cel48S_CD protein in *E. coli* according to published procedure [17]. The Cel48S_CD protein showed low activity toward Avicel, which was similar to that of Cel48S [17]. However, when we further purified the Cel48S_CD protein by gel filtration and observed two fractions with different retention volumes, indicating an impurity of previously purified proteins. Although the two fractions may both refer to Cel48S_CD according to SDS-PAGE analysis, they were significantly different in Avicel hydrolysis activity. Over 80% of purified proteins belonged to the fraction with a lower retention volume (fraction-50, likely Cel48S_CD oligomers) showing a low activity of 8.45 ± 1.13 U/mg. The proteins in fraction-80 with higher activity may represent active Cel48S_CD. Thus, the low Cel48S activity reported in previous studies may be explained by the insufficient purity of the protein, and the activity was significantly underestimated. We further optimized the dialysis and purification processes of the rCel48S_CD inclusion body. However, the fraction referring to inactive protein could not be completely eliminated, and fraction-80 may have contained fraction-50 proteins that could influence the determination of the enzyme activity. This would further influence protein engineering and mutation validation. It is known that Cel48S is a secreted protein in *C. thermocellum* that is assembled extracellularly in the cellulosome. Thus, the insolubility of rCel48S in *E. coli* may be due to the lack of transmembrane step after expression. Because the protein must be in unfolded state for cross-membrane transport, the protein must be correctly refolded extracellularly. Specific chaperonins may also be involved in *C. thermocellum* [50]. We determined to find a way to purify native Cel48S_CD proteins directly from *C. thermocellum* for biochemical or structural analyses. Although the Cel48S protein is typically in high abundance in the extracellular proteins of *C. thermocellum* [25, 26], the Cel48S protein is tightly assembled into the cellulosomal complex via the type I interaction between its dockerin domain and the cohesin domain of scaffoldin protein CipA [27]. Although the liberation of the whole Cel48S protein from cellulosome cannot be accomplished without SDS treatment [42], which may influence its activity, the catalytic domain of Cel48S could be released from the cellulosome in the form of S8-tr component via protease K treatment. However, the purified S8 protein still shows high activity toward xylan, and its purification still requires complicated procedures [14]. Hence, we were determined to release the catalytic domain of Cel48S by inserting a stop codon ahead of the dockerin module in the genome of *C. thermocellum* using a previously developed seamless genome-editing system. A sequence encoding 12 successive histidine residues was also inserted at the C-terminus of the GH domain of Cel48S for one-step purification via Ni^2+^-affinity chromatography. Long poly-histidine tag was used to enhance Cel48S_CD proteins affinity to the chelator on the resin [51]. In addition, because the catalytic region of the GH domain is not close to its C-terminal according to the published rCel48S_CD crystal structure [19], we considered that the linkage of a 12-histidine tag at the C-terminal would not severely affect the activity of the enzyme.

A high-purity catalytic domain of the native Cel48S protein, termed ctCel48S_CD, was obtained from the extracellular proteins of *C. thermocellum* after one-step affinity purification. Unlike rCel48S_CD, the activity of ctCel48S_CD was not dependent on the purification process, as shown by the similar activities among the ctCel48S_CD proteins obtained from three independent cell cultivations and purifications. The purified ctCel48S_CD protein had a high activity of 117.61 ± 2.98 U/mg with the Avicel substrate under assay conditions. The optimal assay conditions for cellulose hydrolysis were similar to those for rCel48S_CD (70 °C, pH 5.7). According to previous reports, the rCel48S protein shows a preference for amorphous cellulose (e.g., PASC) [17, 22], but here, ctGH48 showed a preference for crystalline cellulose Avicel. The rCel48S obtained in a previous study included the dockerin module, which may alter enzymatic properties, the extent to which was not determined. This could also be explained by the impurity of recombinant Cel48S. Based on our results, we assumed that the rGH48 proteins used in previous studies were primarily composed of oligomer-like fractions with low activity. The amorphous cellulose prepared by phosphoric acid-mediated decrystallization had higher accessibility compared with high crystallinity cellulose [52, 53]. Thus, the amorphous cellulose was much easier for the prepared impure r Cel48S protein to hydrolyze than Avicel. The substrate specificity of the purified ctCel48S_CD may reflect the true feature of the exoglucanase Cel48S.

The structure of the ctCel48S_CD proteins showed no posttranslational modifications. A previous mass spectrum analysis of the cellulosome components revealed some potential modifications of rCel48S, including the oxidation of the M290, M451, M552, W266, W493, W469, M298, W472, W595, and Y265 residues [54]. These modifications were likely caused by oxidative damage during sample preparation. The structure of the ctCel48S_CD proteins was obtained without substrate binding. However, a long PEG molecule was observed in the substrate channel, which may mimic the structure of a cellulose chain. Therefore, most residues showed similar conformations as those in previously published structures of rCel48S_CD-substrate complexes. We observed conformational changes of three important residues: the substrate-binding residues R249 and Y431, and the catalytic residue E87. The conformational changes are likely important for substrate binding, product releasing, and cellulose chain movement.

## Conclusion

In this study, we successfully purified the catalytic domain of the native Cel48S protein and ctCel48S_CD, directly from the culture broth of *C. thermocellum* DSM1313. Based on the thorough enzymatic and structural analyses of recombinant Cel48S_CD and native Cel48S_CD proteins, we confirmed that the activity and substrate specificity of Cel48S_CD from *C. thermocellum* were consistent with its importance in the cellulosome. The native Cel48S_CD proteins had no significant posttranslational modification according to mass spectrum and structural analysis. Furthermore, our purification strategy can be used for direct purification of cellulosomal and other secretive proteins from *C. thermocellum*.

## Additional files

**Additional file 1.** Primers used in this study.

**Additional file 2.** Gel filtration chromatograms, activity and relative abundance of rCel48S_CD proteins. After Ni^2+^-affinity purification, the denatured rCel48S_CD proteins were dialyzed against 10 mM Tris-HCl buffer (pH 8.0) containing 100 mM NaCl at 25 °C for 6 h with two buffer changes (b) or for 9 h with three buffer changes (c) and were further purified with a Superdex 200 column (GE healthcare). Two peaks were detected with retention volumes of approximately 50 and 80 mL (fraction-50 and fraction-80, respectively). The relative abundances of fraction-50 or fraction-80 were determined by calculating the proportion of the area of corresponding peak in the total area, i.e., the relative abundance of fraction-50 is Area_fraction-50_/ (Area_fraction-50_ + Area_fraction-80_). The activities of each fraction are shown below the chromatograms.

**Additional file 3.** The theoretical amino acid sequence of ctCel48S_CD with a His_12_-tag at the C-terminal (a) and the molecular weight analysis by HPLC-Q-TOF-MS (b). A linker composed of five glycine is shown in red, and the His_12_-tag is highlighted in yellow. The stop codon is indicated by a asterisk.

**Additional file 4.** Kinetic analysis of ctCel48S_CD. a, Avicel hydrolysis over time. The glucose production was in a linear relationship with the reaction time for 6 h. b, Determination of kinetic parameters of ctCel48S_CD. The reaction lasted for 5 h with initial Avicel concentrations of 1–10 mg/mL. The linear or non-linear fit curves are shown in red, and the corresponding R2 values are given.
