# Supplementary material for: Determination of the native features of the exoglucanase Cel48S from Clostridium thermocellum
Source: Biotechnol Biofuels. 2018 Jan 13;11:6. doi: 10.1186/s13068-017-1009-4 (PMC5766998; doi:10.1186/s13068-017-1009-4)
Supplement: Supplementary file 1 — Additional file 1. Primers used in this study. [file 13068_2017_1009_MOESM1_ESM.docx]

**Table S1 Primers used in this study**

| Primers | Sequence^*^ |
| --- | --- |
| rCel48S-F  rCel48S-R  H_12_TAA-1  H_12_TAA-2 | CTA GCTAGC GGTCCTACAAAGGCACCTAC  CGG ACTAGT AGAAGGAGTACCAGGTACTTTATATG  acc acgcgt **tta**atgatgatgatgatgatgatgatgatgatgatgatg cggccg acc  GGT CGGCCG catcatcatcatcatcatcatcatcatcatcatcat**TAA** ACGCGT GGT |

***** restriction sites and stop codons were underlined and boldfaced, respectively. The His_12_-tag encoding sequences were indicated by lower case letters
