# Supplementary figures and images for: Determination of the native features of the exoglucanase Cel48S from Clostridium thermocellum
Source: Biotechnol Biofuels. 2018 Jan 13;11:6. doi: 10.1186/s13068-017-1009-4 (PMC5766998; doi:10.1186/s13068-017-1009-4)

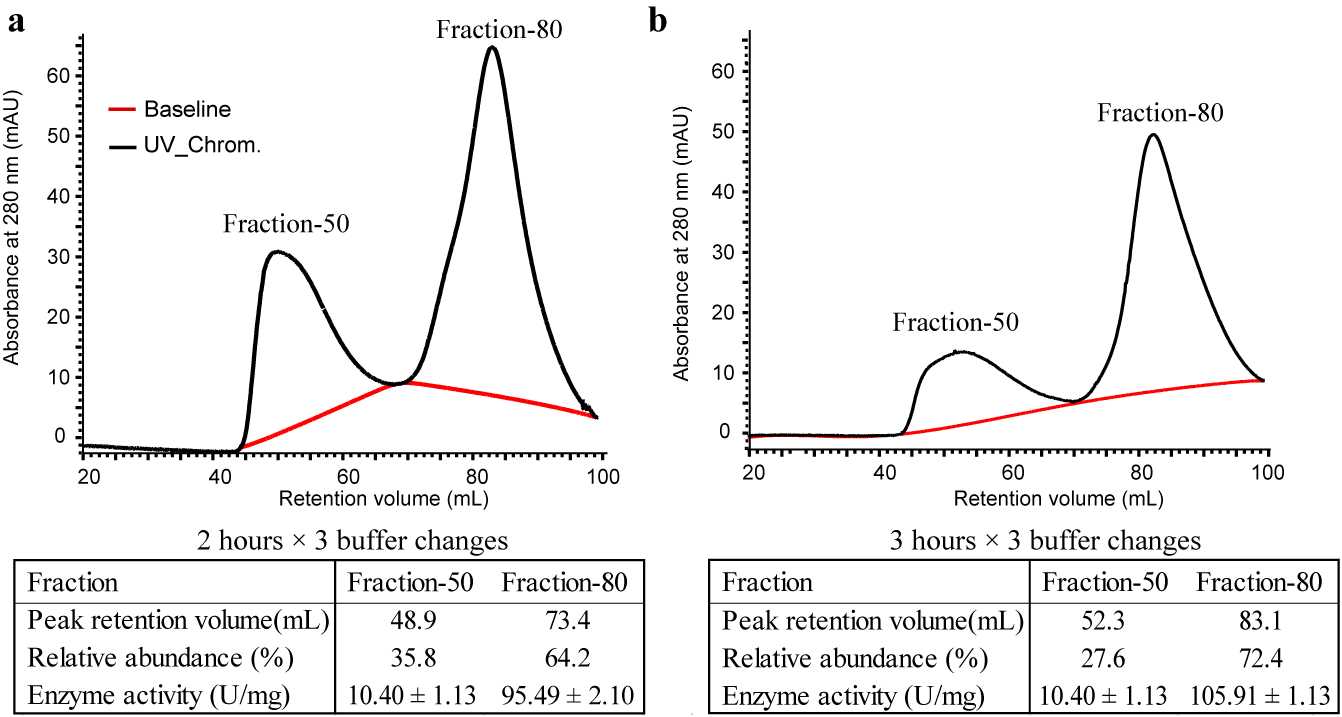

Supplement: Supplementary file 2 — Additional file 2. Gel filtration chromatograms, activity and relative abundance of rCel48S_CD proteins. After Ni2+-affinity purification, the denatured rCel48S_CD proteins were dialyzed against 10 mM Tris-HCl buffer (pH 8.0) containing 100 mM NaCl at 25 °C for 6 h with two buffer changes (b) or for 9 h with three buffer changes (c) and were further purified with a Superdex 200 column (GE healthcare). Two peaks were detected with retention volumes of approximately 50 and 80 mL (fraction-50 and fraction-80, respectively). The relative abundances of fraction-50 or fraction-80 were determined by calculating the proportion of the area of corresponding peak in the total area, i.e., the relative abundance of fraction-50 is Areafraction-50/ (Areafraction-50 + Areafraction-80). The activities of each fraction are shown below the chromatograms. [file 13068_2017_1009_MOESM2_ESM.tif]

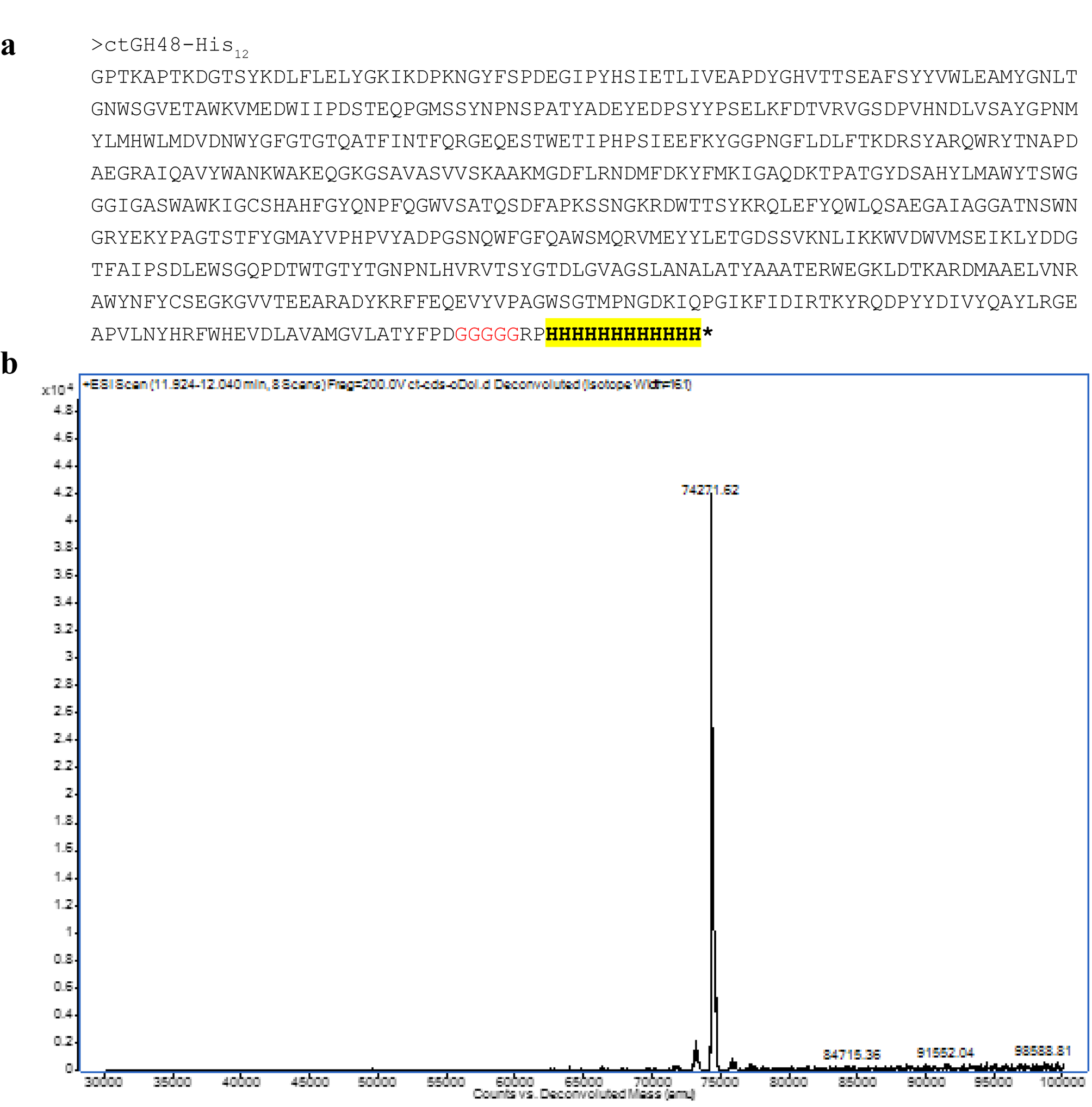

Supplement: Supplementary file 3 — Additional file 3. The theoretical amino acid sequence of ctCel48S_CD with a His12-tag at the C-terminal (a) and the molecular weight analysis by HPLC-Q-TOF-MS (b). A linker composed of five glycine is shown in red, and the His12-tag is highlighted in yellow. The stop codon is indicated by a asterisk. [file 13068_2017_1009_MOESM3_ESM.tif]

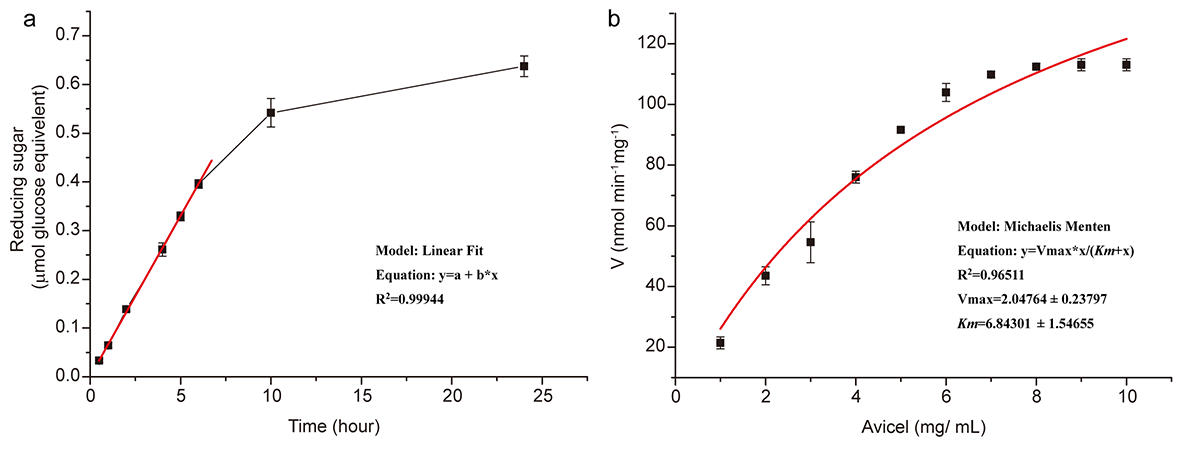

Supplement: Supplementary file 4 — Additional file 4. Kinetic analysis of ctCel48S_CD. a, Avicel hydrolysis over time. The glucose production was in a linear relationship with the reaction time for 6 h. b, Determination of kinetic parameters of ctCel48S_CD. The reaction lasted for 5 h with initial Avicel concentrations of 1–10 mg/mL. The linear or non-linear fit curves are shown in red, and the corresponding R2 values are given. [file 13068_2017_1009_MOESM4_ESM.tif]
